# Lead Extraction: "Drag Through" Technique

**Published:** 2002-10-01

**Authors:** Anoop K Gupta

The goal of extraction techniques of chronic pacemaker and defibrillator leads is to present an approach that is successful in extracting all leads and minimizes or eliminates complications. To extract a chronically implanted intravascular device, the device must be separated from the encapsulating inflammatory tissue. Three ablation techniques are currently used: mechanical, laser and electrosurgical.

Indirect traction is traction applied by an instrument such as a snare passed into the heart, usually through a femoral vein. The lead is entrapped in the snare, and pulling or pushing applies traction [1,2]. The safety of this technique is enhanced by the ability to avoid those problems associated with the binding sites in the superior veins and right atrium. Indirect traction is more successful than direct traction. Countertraction is a method of safely extracting an electrode entrapped in fibrous tissue at the electrode-myocardial interface. It is defined as the countering of the traction on the lead by a sheath. A sheath of slightly larger diameter is passed over the lead to a point about 1 cm from the heart wall. Traction is applied on the lead, pulling the myocardial wall to the edge of the sheath, which counters the traction. Because only the scar tissue is present between the sheath and the heart wall, cardiac tissue is not in jeopardy.

The transvenous approach through the femoral vein requires a special sheath set that function as an introducer, as a workstation for manipulation of snares and as counter tractions sheaths. The Byrd Femoral Workstation set consists of an introducer needle, guide wire, 16-F workstation, 11-F tapered dilator, 11-F telescoping sheath, a cook deflection snare, and a Dotter basket snare. The needle's Eye snare (cook vascular Inc., Leechburg, PA) is another apparatus that can be inserted through the Byrd Femoral Workstation.

Countertraction is applied by means of the implant vein through the superior vena cava (SVC) approach, and indirectly, by means of the femoral vein through the IVC approach [3,4]. The rationale for using the SVC approach is greater speed, less fluoroscopic exposure, and a high success rate for undamaged leads implanted less than 6-8 years. The IVC approach is more versatile approach. It is procedure of choice for broken or cut leads floating freely in the veins, heart, or pulmonary artery and for leads passing through occluded veins. A drawback to femoral approach lead extraction is no vascular access for new system implantation, which is particularly relevant in-patient with subclavian or brachiocephalic vein occlusion. The incidence of venous occlusion following permanent pacemaker implantation is approximately 12% [5].

Staniforth et al. in this issue has described a femoral drag through technique for extraction of pacemaker and defibrillator leads and subsequent replacement through the same access. They performed extraction in 14 patients using Byrd Femoral Workstation in 6 patients and needle eye snare in 8 patients. The complete success rate in their study was 91% lead (86% patients). Klug et al. in their study has shown 87.2% success with needle's eye snare for femoral approach. Countertraction in 39 patients with a total of 82 leads [6]. The failure from this approach is related mainly due to impossibility of advancing the 16Fr long sheath through the right and left iliac veins and lead totally excluded from the venous flow.

Staniforth et al. has demonstrated an alternative method for lead replacement from the same approach in patients with vein occlusion using the non-functioning leads and this avoids the possibility of bilateral subclavian occlusions, failure to cross superior vena cava or the need of epicardial or femoral implant. Laser assisted approach is the other method for replacing leads from the same approach [7]. The femoral drag through approach is cheaper more safe and can be performed under local anesthesia.

Even with the latest advances, successful lead extraction via percutaneous techniques still cannot be achieved in up to 2% of procedures. The femoral drag through procedure is relatively safe and efficacious in comparison to the other methods.
